# Development and Characterization of a Novel Peptide-Loaded Antimicrobial Ocular Insert

**DOI:** 10.3390/biom10050664

**Published:** 2020-04-25

**Authors:** Eleonora Terreni, Susi Burgalassi, Patrizia Chetoni, Silvia Tampucci, Erica Zucchetti, Roberta Fais, Emilia Ghelardi, Antonella Lupetti, Daniela Monti

**Affiliations:** 1 Department of Pharmacy, University of Pisa, 56126 Pisa, Italy; eleonora.terreni@farm.unipi.it (E.T.); susi.burgalassi@unipi.it (S.B.); patrizia.chetoni@unipi.it (P.C.); silvia.tampucci@unipi.it (S.T.); erica.zucchetti@phd.farm.unipi.it (E.Z.); 2 Department of Translational Research and New Technologies in Medicine and Surgery, University of Pisa, 56126 Pisa, Italy; roberta.fais91@live.it (R.F.); emilia.ghelardi@med.unipi.it (E.G.); antonella.lupetti@med.unipi.it (A.L.)

**Keywords:** hLF 1-11, freeze-drying, ocular insert, peptide, antimicrobial activity, AMPs, ocular infection, mucoadhesive polymers

## Abstract

Infectious ocular keratitis is the leading cause of blindness worldwide. Bacterial resistance to classical pharmacological treatments raised the interest of researchers towards antimicrobial peptide (AMP)-based therapy. hLF 1-11, a synthetic antimicrobial peptide derived from the N-terminus of human lactoferrin, proved effective against different bacteria and yeast but, like all proteinaceous materials, it is unstable from chemical, physical, and biological points of view. In this study, new freeze-dried solid matrices containing mucoadhesive polymers were prepared and characterized in terms of rheology, hydration time, bioadhesion, drug content, and in vitro release. The formulation HPMC/T2/HA/hLF 1-11_fd_ was selected for the delivery of hLF 1-11, since it showed good drug recovery and no chemical degradation up to at least 6 months (long-term stability). Furthermore, the HPMC/T2/HA/hLF 1-11_fd_ matrix allowed for the release of the drug in a simulated physiological environment, linked to an optimal hydration time, and the peptide antimicrobial activity was preserved for up to 15 months of storage, a very promising result considering the chemical liability of proteinaceous material. For its properties, the freeze-dried matrix developed in this study could be a good platform for the delivery of antimicrobial peptides in the precorneal area to treat infectious phenomena of the ocular surface.

## 1. Introduction

Infectious keratitis is the leading cause of blindness worldwide. Delayed or inadequate treatment of this infection can lead to corneal perforation, which can evolve into endophthalmitis and complete vision loss. The WHO has estimated that corneal ulcers are responsible for 1.5–2.0 million new cases of monocular blindness every year [1,2,3,4,5]. The first-line treatment of bacterial keratitis is represented by topical antibiotic drops [3,6]. However, the spread of bacterial drug resistance raised the interest of researchers into the development of alternative therapeutic strategies based on antimicrobial peptides (AMPs) [7]. AMPs are small peptides with broad-spectrum activity against bacteria, fungi, and viruses.

Lactoferrin is an important component of the non-specific defense against infections and excessive inflammation [8,9] and is present at high concentrations in mucosal secretions, such as tears and milk [8,9,10,11]. A synthetic antimicrobial peptide derived from the N-terminus of human lactoferrin (hLF 1-11, GRRRRSVQWCA, 1374 Da) was shown to be more powerful than the whole lactoferrin against different bacteria and yeast both in vitro and in vivo [12,13,14,15,16]. Indeed, hLF 1-11 was very effective in systemic infections caused by fluconazole-resistant *Candida albicans*, multidrug-resistant *Staphylococcus aureus*, and *Acinetobacter baumannii* strains [12,13,14,17,18,19,20]. Noteworthy, as previously reported, the doses of hLF 1–11 required for antifungal effects in mice with a disseminated *C. albicans* infection were much lower than those required in in vitro experiments [14]. Possible explanations for these observations include synergistic/additive effects between this peptide and host-derived antifungal factors, such as endogenous antimicrobial proteins/peptides and reactive oxygen intermediates [21]. hLF 1-11 showed immunomodulatory activities, such as regulation of monocyte differentiation, enhancement of TLR-mediated immune response in monocytes, and inhibition of the myeloperoxidase activity [14,22,23,24,25,26,27]. Moreover, the cationic residues (-RRRR-) of hLF1-11 are flexible, and thus suitable for interaction with the anionic bacterial membrane. In fact, the hydrophobic region, which is positioned approximately perpendicular to the cationic residues, enabled the peptide to bind to the cytoplasmic membrane, highlighting its structural selectivity between bacterial and eukaryotic membranes [28].

One of the main factors that challenges the delivery of peptides is their physical, chemical, and biological instability due to denaturation, adsorption, aggregation, and precipitation typical of protein-like drugs. In addition, conformational changes due to pH, temperature, or high salt concentration may lead to the inactivation of the proteinaceous material in ocular tissues [29]. Therefore, the stability issue is the main challenge in the development of peptide-based formulations. An approach to overcome the problem of both instability and rapid elimination of these molecules from the administration site could be the development of a solid dosage form able to protect the drug and maintain the formulation in the delivery site by producing a prolonged release.

With the aim of finding a good system able to overcome the instability of peptides and suitable for the application in the precorneal area of the eye, in the present study, solid matrices of different compositions regarding both polymeric materials (hydroxypropyl methylcellulose and sodium hyaluronate as mucoahesive polymers) and other excipients such as mannitol, a bulking agent, and trehalose, a lyoprotectant, were produced by freeze drying. To begin, the matrices were loaded with vancomycin as a model molecule for its similarities with hLF1-11 in term of MW (1485.74 Da) and chemical nature (glycopeptide antibiotic). The formulation that showed no degradation phenomena during the manufacturing process, with the best mucoadhesive characteristics and most suitable release profile, was selected to be loaded with the hLF 1-11 target peptide. This final formulation was tested for both long-term chemical stability (for up to 6 and 15 months of storage) and antimicrobial activity against *Staphylococcus epidermidis*, the leading cause of bacterial endophthalmitis and frequently associated with infectious keratitis worldwide [30].

## 2. Materials and Methods 

### 2.1. Chemicals

The following materials were used: hydroxypropyl methylcellulose, HPMC (MethocelTM K4M Premium EP; Colorcon Ltd., Dartford, UK); sodium hyaluronate, HA (1000–1800 kDa, Phylcare^®^, Biophil, Italy); mannitol (Fine Foods NTM, Italy); trehalose (Hayashibara Co., Ltd., Okayama, Japan); hog gastric mucin, HGM (Carl Roth GmbH & Co. KG, Karlsrube, Germany); and vancomycin hydrochloride, VA (European Pharmacopoeia; Sigma-Aldrich, Italy).

The synthetic peptide corresponding to residues 1-11 (GRRRRSVQWCA) of hLF, further referred to as hLF 1-11, was purchased from Peptisyntha (Brussels, Belgium). The purity of this peptide exceeded 99%, as determined by reverse-phase high performance liquid chromatography (RP-HPLC). Peptide stocks at a concentration of 10 mM in 0.01% acetic acid (pH 3.7) were stored at −20 °C and diluted to the desired concentration before use.

Phosphate buffer saline solution (PBS, 66.7 mM, pH 7.4, isotonic) consisted in 1.84 g/L NaH_2_PO_4_·H_2_O, 7.57 g/L Na_2_HPO_4_, and 4.4g/L NaCl.

Artificial tear fluid (ATF, pH 7.4) was composed by MgCl_2_ (4.75 mg/100 mL), CaCl_2_ (7.92 mg/100 mL), KHCO_3_ (260 mg/100 mL), and NaCl (754 mg/100 mL).

All other reagents were analytical grade, and Milli-Q water was used throughout.

### 2.2. VA-Loaded Matrices 

#### 2.2.1. Preparation and Characterization of the Freeze-Dried Inserts 

Lyophilized matrices, whose compositions are reported in Table 1, were prepared by freeze-drying (VirTis Wizard 2.0) appropriate aliquots of the polymeric dispersions.

The polymers were dispersed in MilliQ water and maintained under stirring at room temperature for 12 h. Mannitol and trehalose were used as bulking and cryoprotectant agents, respectively, in a percentage ranging from 1 to 2 wt % of total dispersion. Vancomycin was added to the polymeric vehicle, under stirring conditions, before freeze-drying: 500 μL of the drug solution (1.0 mg/mL) was added to 3.25 g of the polymeric dispersion to obtain 25 matrices (one blister) (150.0 μL/insert). Each matrix contained 0.02 mg of VA.

For bulk studies, 17.0 g of the same dispersion were placed in PTFE Petri dishes (8.6 cm diameter). All formulations underwent the following freeze-drying cycle under controlled conditions: Freezing: pressure 400 torr; temperature −38 °C; rate 0.6 °C/h; extra freeze time 120 min. Primary drying: pressure 100 torr; temperature −38 to 0 °C; rate 2.1 °C/h. Secondary drying: pressure 50 torr; temperature 0 to 25 °C; rate 5.0 °C/h; extra drying 27 °C for 60 min) [31].

The freeze-dried matrices were stored in desiccators until use. The matrices compositions are summarized in Table 1. Six randomly selected matrices of two different batches were weighed and three of them were analyzed in terms of drug content to verify both the reproducibility and the influence of the preparation method on drug stability. Each matrix was re-dispersed in 1 mL of PBS and stirred until complete matrix re-dispersion; the dispersion was then analyzed by RP-HPLC after the appropriate dilution. Drug recovery (%) was calculated as: (amount of drug recovered/initial amount of drug used) * 100.

Rheological behavior of the polymeric dispersions before and at the end of the freeze-drying process and after matrix re-dispersion was determined at 25 °C by a Rheostress RS 1 (Haake, Germany) equipped with coaxial cylinders (Z40 and Z41) at shear rates (D) ranging from 0 to 250 s^−1^. The polymeric dispersions exhibited a pseudoplastic flow described by the Ostwald-de-Waele power law: τ = KD^N^ Equation (1) and their apparent viscosity, η′, was calculated for D = 1 s^−1^ [32].

The time required to obtain a complete re-dispersion of matrices in the artificial tear fluid (ATF) was determined and reported as water sorption time (WST). WST was qualitatively monitored by digital microscope (Dino-lite Pro, ANMO, Taiwan): a freeze-dried polymeric matrix was placed into a Petri-dish standing on a dark background used as contrast; the amount of solvent removed during the freeze-drying process was added to the matrix to reconstitute it to the original weight [31]. The endpoint was established when the bottom of the Petri-dish was again clearly visible.

#### 2.2.2. Mucoadhesion Test

Mucoadhesive properties of the lyophilized matrices, HPMC/T2/VA and HPMC/T2/HA/VA, were evaluated by measuring their work adhesion (W) on a mucous surface by a tensile (detachment) test using the method described by Burgalassi et al. [32]. Briefly, 125 μL of a 28.0 wt % aqueous dispersion of HGM uniformly spread on wet filter paper disks of 12 mm diameter tightly secured to both cell cylindrical sections of the apparatus were superficially dried for 5 min by blowing on them with cold air. Then, 50 microliters of the sample under study (freeze-dried matrices re-dispersed separately with 30 μL of water) were thinly layered onto the upper mucous surface. The lower cell section was slowly raised and put into contact with the sample; after 90 s of contact, the cell sections were moved away at a constant speed (1.25 mm/min) up to complete separation. Analysis of the resulting force versus distance curves (work of adhesion, W) was performed using Prism software v.7.0a (GraphPad Software Inc., La Jolla, CA, USA). All W values were normalized with respect to the adhesion area. The evaluation included the calculation of the mean of six determinations and standard errors; the differences were evaluated by the one-way ANOVA statistical analysis (Prism v. 7 software). Differences were considered statistically significant at *p* < 0.05.

#### 2.2.3. DSC Analysis 

Differential scanning calorimetry (DSC) measurements were carried out in triplicate using a Perkin Elmer differential scanning calorimeter (DSC 6, PerkinElmer, Italy) on raw materials (p_rm_) and the physical mixtures of the matrix components (p_mix_), while maintaining the same polymer(s)/cryoprotectant agent(s) ratio as that of the matrix, and freeze-dried matrix (fd). The equipment was calibrated with purified indium and zinc (99.9%). Samples (1.5–2.0 mg) were placed and sealed in a flat-bottomed aluminum pan and heated at a constant rate of 5 °C/min under nitrogen purge gas at a rate of 20 mL/min. The specimens were heated from 30 °C to 240 °C. The thermogravimetric curves were recorded with Pyris Instrument Managing Software (Version 3.8, Perkin Elmer, Milan, Italy) and analysis was performed using IgorPro^®^ software (Version 7.0, WaveMetrics Inc., Poland, OR, USA).

### 2.3. hLF 1-11-loaded Matrices

#### 2.3.1. Preparation and Physicochemical Characterization 

The results obtained with vancomycin led to the selection of the HPMC/T2/HA formulation as the most promising for ocular delivery of the hLF 1-11 peptide. The matrix HPMC/T2/HA/hLF_fd_ was prepared by adding to the polymeric dispersion the appropriate amount of a 0.01% glacial acetic acid solution of hLF 1-11 peptide to obtain 0.02 mg of drug in each matrix after the freeze-drying process. The content of drug was quantitatively determined after re-dispersion of the matrix in PBS. To verify the influence of trehalose, as a cryoprotectant agent, the matrix HPMC/HA loaded with hLF 1-11 (HPCH/HA/hLF_fd_) was also prepared and used as a reference.

Long-term stability of the drug in the HPMC/T2/HA/hLF_fd_ matrix was also monitored by determining the peptide amount after 2, 3, and 6 months of matrix storage, and drug recovery in percentage was determined by HPLC analysis. The samples were stored in a desiccator (with calcium chloride) at room temperature and protected from light.

More specifically, analyses were performed immediately after dissolution of the matrix in 1 mL of PBS. As reference standards, hLF 1-11 powder was dissolved in PBS and progressively diluted with the same solvent. The standard solutions were analyzed immediately after peptide solubilization.

#### 2.3.2. In Vitro Susceptibility Testing

The minimum inhibitory concentration MIC of hLF 1-11 against *Staphylococcus epidermidis* ATCC 35,984 was evaluated by the microdilution method in round-bottom polystyrene 96-well microtiter plates (Corning Costar, Sigma). This assay was performed in RPMI 1640 containing MOPS and glucose (Merlin Diagnostika GmbH, Germany) further diluted by 1/4 in NaPB. hLF 1-11 was tested in the concentrations ranging from 0.12 to 128 μM. Briefly, the two-fold dilutions of hLF 1-11 were set up in 100 μL of RPMI/MOPS broth diluted by 1/4 and then an equal volume of the mid-log phase bacterial suspension in the same medium was inoculated into each well of the plate at a final concentration of approximately 1 × 10^6^ colony-forming units (CFU)/mL. Sterility control wells, containing the medium alone, were included in each plate. After 18–24 h incubation at 37 °C, the MIC of the peptide was defined on the basis of the turbidity of the wells as the lowest concentration of the agent that produced the complete inhibition of visible growth. Next, 100 μL of the suspensions obtained by the MIC assay at the MIC concentration of hLF 1-11 and concentrations of hLF 1-11 higher than the MIC value were inoculated into blood agar plates to evaluate the minimal bactericidal concentration (MBC) of hLF 1-11 against *S. epidermidis*. Plates were incubated at 37 °C for 24 h to determine the number of CFU. The MBC was defined as the lowest concentration of hLF 1-11 killing ≥ 99.9% of viable microorganisms after 24 h incubation. All tests were performed in triplicate.

In vitro hLF 1-11 release from the the HPMC/T2/HA/hLF_fd_ matrix was assessed in a bacterial killing assay performed against *S. epidermidis* ATCC 35984. The matrix containing 10 µg of hLF 1-11 was suspended in 200µL of 10 mM sodium phosphate buffer (NaPB, pH 7.4) supplemented with 2% glucose solution in a glass micro-tube 1 h before starting the experiment, to allow complete dissolution. To evaluate the hLF 1-11 release from the matrix in vitro, killing assays were performed in polystyrene, flat-bottomed, 96-well microliter plates (final volume 100 µL/well). *S. epidermidis* suspensions were prepared at 2 × 10^6^ cells/mL in NaPB supplemented with 2% glucose (50 µL/wells) and different volumes of hLF 1-11 matrix solution were tested (20, 10, 5, 2,5, 0 µL). At the same time, hLF 1-11 solutions at different concentrations (6.4, 3.2, and 1.6 µM) were tested in the same conditions in order to compare the antibacterial activity of the matrix-peptide with the peptide alone. After 1 h of incubation at 37 °C, bacterial suspensions were diluted with PBS and plated onto Luria Bertani (LB) agar plates. After incubation at 37 °C for 24 h, colony-forming units (CFUs) were counted. Five independent experiments were performed. Data were expressed as the mean ± standard error of the mean (SE). Results were evaluated by one-way ANOVA statistical analysis (Prism v. 7 software). The measured values were considered statistically different when *p* < 0.05. The antimicrobial activity of the selected hLF1-11 loaded matrix (HPMC/T2/HA/hLFfd) was evaluated immediately after the preparation of the formulation and at 1, 2, 6, and 15 months. The obtained matrices were stored at room temperature in a desiccator (with calcium chloride) and protected from light until further use.

### 2.4. In Vitro Drug Release Test

In vitro release of drugs (VA or hLF 1-11) from the freeze-dried matrices was investigated by using vertical diffusion cells (Hanson, Chatworth, CA) with effective diffusional area of 0.636 cm^2^. A Durapore^®^ PVDF membrane (25 mm diameter, 0.45 μm) was placed between the receiving and the donor compartments. The receptor medium was PBS (4.0 mL), kept at 32 °C and stirred at 400 rpm. The donor phase consisted of a lyophilized matrix soaked with 30 μL of MilliQ water to simulate the physiological conditions. The release system was sealed to avoid any evaporation phenomenon. Every 30 min in the case of VA or every hour for *hLF 1-11*, the receiving phase was completely withdrawn for the analysis and replaced with fresh buffer. The amount of released drug was determined by RP-HPLC. All experiments lasted 4 h and were performed in triplicate.

### 2.5. Analytical Methods 

The quantitative determination of the drugs under study was carried out by HPLC analysis. The apparatus consisted of a Shimadzu LC-20AD system with a UV SPD-10A detector equipped with an auto sampler SIL-10AD VP and a computer integrating system. Specific characteristics of the analytical methods used are reported in Table 2. The amount of each drug in the samples was determined by comparison with an appropriate standard curve. In the case of VA, the drug was dissolved in MilliQ water and diluted in PBS (concentration range: 0.052–2.06 μg/mL) whereas peptide (hLF 1-11) stock solution (1 mg/mL) in 0.01% glacial acetic acid [14] was progressively diluted in PBS to obtain a concentration range of 1.0–15.0 μg/mL. The correlation coefficients (r^2^) were 0.9996 and 0.978 for VA and hLF 1-11, respectively.

## 3. Results and Discussion

### 3.1. VA-Loaded Matrices

#### 3.1.1. Preparation and Characterization of the Freeze-Dried Inserts Containing VA

The freeze-drying technique allows for the better handling of thermo-labile active pharmaceutical ingredients (API) by eliminating water at low temperatures to ensure long-term stability of the final product [33,34] and production of matrices with a rapid hydration/solubilization when they come in contact with the tear fluid of the eye cul-de-sac.

Hydroxypropyl methylcellulose (HPMC) and hyaluronic acid (HA) were chosen as mucoadhesive polymers, as they are widely used in the development of ophthalmic dosage forms. All the dispersions contained 1 wt % of total polymer as HPMC or a mixture of HPMC and HA (ratio 9:1). Mannitol was used as the crystalline bulking agent to obtain a uniform cake appearance [35]. Moreover, trehalose (ratio 1:2) was introduced as a cryoprotectant and lyoprotectant agent to stabilize the peptide during the freeze-drying process, replacing a part of the mannitol, since it is known that the crystalline modification of mannitol during the lyophilization process might negatively impact the storage stability of proteins [36,37].

**Figure 1:**
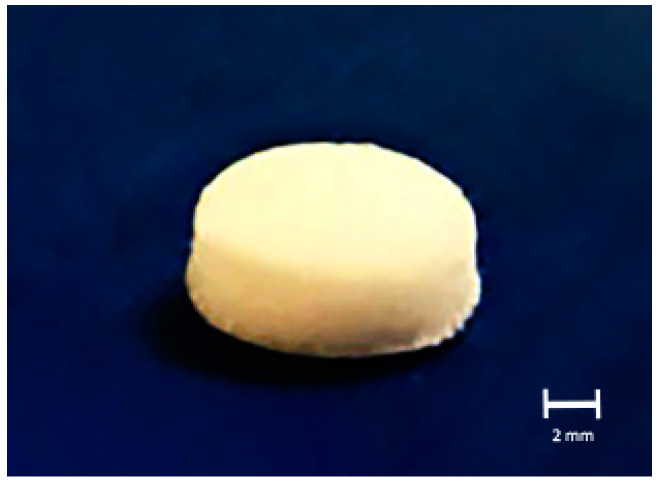
Freeze-dried matrix.

The integrity of the components of freeze-dried matrices was visually evaluated in terms of cake collapse, volume, melt-back, puffing, texture, cracking, and color changes [38]. As shown in Figure 1, good cake quality was obtained for all developed matrices (diameter: 7.88 mm, thickness: 2.30 mm, area: 48.73 mm^2^), with no fractures or cake collapse.

Coefficients of variation [CV = (SD/mean) * 100] were calculated for the weights of the matrices belonging to two different batches for each developed formulation (Table 3). CV ranged between 1.37% and 2.04% for a mean weight of the matrices between 5.67 and 5.85 mg. These low CV values, in accordance with the indications in the European Pharmacopoeia, suggest a good reproducibility of the preparation method.

To understand the influence of the freeze-drying conditions on the stability of the final product, the rheological characteristics were checked before and after lyophilization. All formulations showed pseudoplastic behavior and Table 4 summarizes the apparent viscosity values, in mPa*s at D = 1 s^−1^, taking into account the weaker interactions of the macromolecules when the fluid is at rest, such as during the formation of adhesive bonds [32].

The HPMC/VA_disp_ starting dispersion showed a viscosity of 227.7 ± 11.82 mPa*s. The presence of high molecular weight HA (1000–1800 kDa) produced a predictable increase in viscosity: HPMC/HA/VA_isp_ gave the highest viscosity value (528.3 mPa*s) [39]. The addition of trehalose instead of a part of mannitol showed a small increase in viscosity, which became more marked when hyaluronic acid was present (HPCH/T2/HA/VA_disp_, 321.2 mPa*s). After freeze-drying, the viscosity of the dispersions obtained from the reconstituted matrices tended to decrease, but only in the case of HPMC/HA/VA_fd_ was the difference statistically significant (351.7 ± 23.76 vs. 528.3 ± 2.00 mPa*s after and before freeze drying, respectively). This behavior could be attributable to a partial depolymerization of HA during the lyophilization process [40]. This phenomenon seemed to disappear after partial replacement of mannitol with trehalose (HPCH/T2/HA/VA_fd_), directing the choice towards this formulation.

The role of the lyophilization process on the stability of drugs was verified by determining the VA content of the final matrices. Drug recovery values (%), summarized in Table 5 as the mean of three determinations ± SE, ranged between 91.39% and 100.2%, depending on the formulation. Drug recovery from the matrices containing mannitol was about 92%; the partial replacement of mannitol with trehalose, in a ratio 2:1:1 (trehalose/polymer(s)/mannitol), proved to be an optimal strategy to stabilize protein-like drugs with an increase of drug recovery ranging from 96% to 100.2% for HPCH/T2/VA and HPCH/T2/HA/VA formulations, respectively. It is known that sugars should be able to interact with the protein-like drug during the freeze-drying process to maintain the protein’s native conformation and reduce both local and global mobility during the storage of the final product. In particular, small sugars, such as disaccharides, should be able to reduce the local mobility, since they are less inhibited by steric hindrance due to their better ability to form interactions with proteins [41].

The ability of the matrices to rehydrate once applied in vivo was evaluated by the determination of WST. Trehalose-based matrices showed shorter WST between 60 and 120 sec for HPMC/T2/HA/VA_fd_ and HPMC/T2/VA_fd_, respectively. On the other hand, matrices HPMC/VA_fd_ (WST: 12 min) and HPMC/HA/VA_fd_ (WST: 14 min) needed 10 times longer than trehalose-based matrices to rehydrate. The replacement of mannitol with trehalose, highly soluble in water (50.8 vs. 23.8 wt % at 20 °C [42]) appeared to facilitate the interaction with the aqueous medium. In addition, mannitol and sugars (as the sucrose, structurally similar to trehalose) led to different porosity in the freeze-dried material: The former led to large and elongated clusters, while the latter produced small and circular cavities. This can result in a slightly quicker water uptake over time for sugar-based lyophilized polymers than those containing only mannitol [43]. Moreover, increasing the additives amount in the formulation with respect to the polymer quantity, as in this study in which the additive(s)/polymer(s) ratio was 3:1, caused faster water medium permeation into the matrix [44]. Clearly, the WST value can be used as an indicator of the in vivo matrix rehydration time, even if the experiment in vitro was performed in stagnant conditions [31].

#### 3.1.2. Mucoadhesion Test

Tests were performed by re-dissolving the matrices with a volume of 30 μL of water. The work of adhesion, erg/cm^2^, for HPMC/VA_fd_ was 327.0 ± 10.67 erg/cm^2^ and tended to increase when a part of the HPMC was replaced with HA (HPMC/HA/VA_fd_ = 378.7 ± 21.56 erg/cm^2^), confirming the data in the literature about the mucoadhesive properties of hyaluronic acid, even if the differences were not statistically significant (*p* > 0.05). Moreover, these W values were statistically different from those obtained by putting into contact the two surfaces of mucin to determine mucin–mucin interactions (cohesion work: 244.0 ± 14.65 erg/cm^2^), demonstrating that the polymer/mucin adhesion and not the mucin/mucin cohesion was measured. Therefore, lower values than 244.0 ± 14.65 erg/cm^2^ would be found if the interaction/interpenetration between mucin and polymer molecules did not happen.

#### 3.1.3. DSC Analysis

**Figure 2:**
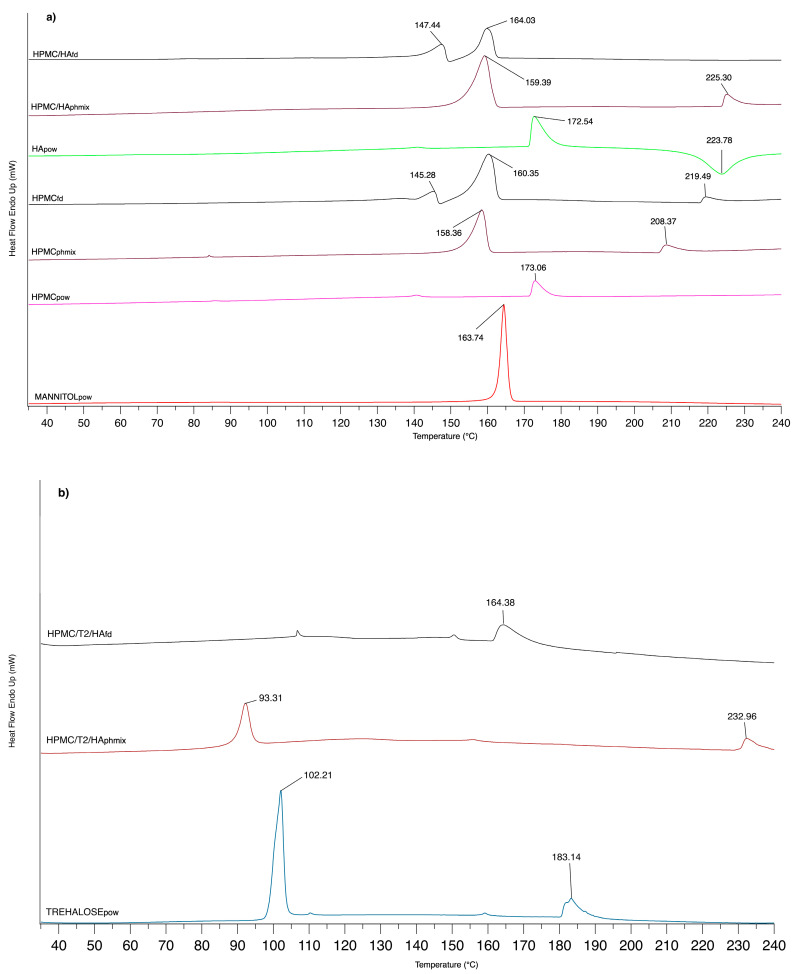
DSC thermograms of (**a**) mannitol, hydroxypropilcellulose (HPMC), hyaluronic acid (HA) powder (*pow*), physical mixture (*pmix*), and freeze-dried matrix (*fd*) of HPMC/HA (total polymer concentration 25 wt %; mannitol 75 wt %) and (**b**) trehalose, physical mixture (*pmix*), and freeze-dried matrix (*fd*) of HPMC/T2/HA (total polymer concentration 25 wt %; trehalose 50 wt %; mannitol 25 wt %).

Influence of the lyophilization process on the components of the formulations under study was verified by DSC analysis. The thermal transitions are graphically reported in Figure 2. At about 173 °C, HPMC_pow_ and HA_pow_ polymers showed a broad endothermic peak (more intense for HA_pow_). In the case of HA, next to the transition at 172.54 °C ascribable to the dehydration process, there appeared an exothermal transition at 223.78 °C for the degradation phenomenon [45]. DSC of pure mannitol exhibited a melting peak at 163.74 °C, a value very close to that reported in the literature [44].

An interaction between HPMC and mannitol seemed to exist, since a widened peak to 158.36 °C, and a new endothermic transition onset at 208.35 °C were observed when the HPMC physical mixture was evaluated. In the case of freeze-died matrices (HPMC_fd_), the lyophilization process appeared to modify the amorphous state, a typical feature of HPMC, since another thermal peak appeared at 145.28 °C for HPMC_fd_. The replacement of a part of HPMC with HA gave similar results for both the physical mixture and freeze-dried matrix.

When part of mannitol had been replaced with trehalose in the physical mixture (HPMC/T2/HA_phmix_), a transition peak at 93.31 °C appeared. Thermogram of pure trehalose revealed two peaks at 102.21 °C (sharp) and at 183.14 °C (wider); the first peak is attributable to the melting of the crystalline part of the dehydrated trehalose, while the latter peak can be associated with the melting of its anhydrous counterpart, which corresponds to the β-form of anhydrous trehalose [46]. The presence of trehalose appeared to influence the interaction with polymers more markedly than mannitol, probably in term of polymer structure order [43]. Intermolecular interactions between sugars and polymers could be the cause of this trend, as demonstrated by Imamura et al. (2010) who studied how Tg values changed when sucrose was combined with, for example, polysaccharides [47].

HPMC/T2/HA_fd_ matrices showed a thermal transition at 164.38 °C, as was the case of the same matrix without trehalose (HPMC/HA_fd_), while the introduction of trehalose appeared to modify the peak at 147.44 °C. The change in the thermal profile could suggest a close interaction among all components of the formulation.

#### 3.1.4. In Vitro Drug Release Studies

**Figure 3:**
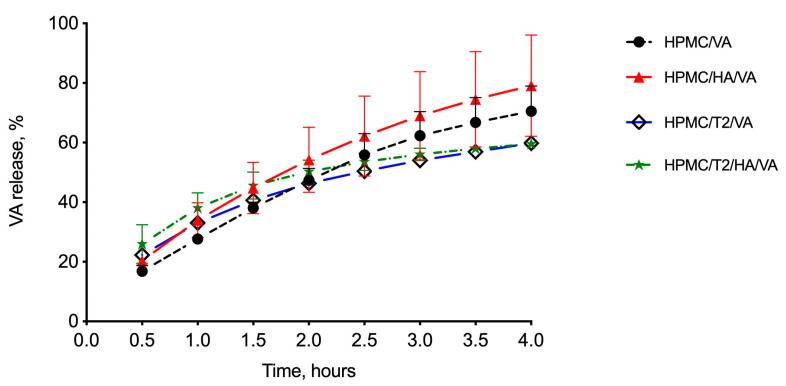
In vitro release profile of vancomicyn (VA) in phosphate buffer saline (PBS) from the freeze-dried matrices under study based on hydroxypropylcellulose (HPMC) alone or in mixture with hyaluronic acid (HA) without (HPMC/VA, HPMC/HA/VA) and with (HPMC/T2/VA, HPMC/T2/HA/VA) trehalose. Data represent mean ± standard error (*n* = 3).

The matrices under study were subjected to in vitro release studies using vertical diffusion cells (VDC); the amount of released drug through the period of the experiment (4 h) was determined by RP-HPLC. All the formulations showed the same drug-release profile (Figure 3, drug released % vs. time): an initial linear trend followed by a slight decrease in the amount of released glycopeptide. In the matrices containing trehalose, this phenomenon was more markedly visible. At the end of the experiment, the percentage of VA released from the matrices was 70.54% ± 8.45% for the reference matrix HPMC/VA_fd_, 79.11% ± 17.02% for HPMC/HA/VA_fd_, 59.79% ± 1.51% for HPMC/T2/VA_fd_, and 59.54% ± 0.94% for HPMC/T2/HA/VA_fd_. Trehalose-containing matrices initially appeared to have a greater release, probably due to faster hydration as demonstrated by the value of WST (1–2 min vs. 12–14 min for the formulation with and without trehalose, respectively); afterwards they showed a slower release.

Mannitol appeared to lead to a faster dissolution, promoting the release rate of the glycopeptide; channel formation into the matrix due to the solubilization of mannitol could facilitate the entry of medium, matrix polymer(s) swelling, and consequent drug dissolution [44]. When in contact with the hydration medium, matrices produced a viscous gel through which the peptide had to diffuse; a delay of drug release could depend on the type of polymeric structure. The addition of trehalose could change the polymer gel structure, by further entrapping the protein-like drug and slightly reducing its diffusion from the donor (matrix) to the receiving phase.

### 3.2. hLF 1-11-Loaded Matrices

#### 3.2.1. Preparation and Physicochemical Characterization

From the preliminary studies described above and carried out with the model drug, the HPMC/T2/HA formulation appeared to be the most promising for the delivery of the antimicrobial peptide hLF 1-11 in terms of matrix chemico-physical characteristics, mucoadhesion, ability to preserve the integrity of the drug during the freeze-drying process, and good drug-release profile.

HPMC/T2/HA/hLF 1-11 matrices were prepared by freeze drying. We evaluated the matrix integrity/alteration visually and by monitoring the changes in peptide content over different storage times by HPLC. The results were compared with the same formulation without trehalose (HPMC/HA/hLF 1-11) used as a reference to confirm its cryoprotectant activity towards the target drug. A good cake quality was obtained, with no fractures or cake collapses, as also seen with vancomycin. As shown in Table 6, the matrix containing trehalose (HPMC/T2/HA/hLF1-11_fd_) had a greater peptide recovery (86.74% ± 1.83 %) than the formulation containing only the bulking agent and polymers (HPMC/HA/hLF1-11_fd_: 68.38% ± 4.43 %), as previously found for the VA-loaded matrix and in a previous work for freeze-dried matrices containing antibodies [31]. The reduction in the percentage of drug recovery compared to the vancomycin-loaded matrices suggests a greater instability of this protein molecule. It is important to point out that this molecule has to be handled with care, because it is subjected to adsorption phenomena on the materials/equipment used in the laboratory (glass, plastic, etc.) [48], and this could give false results. Therefore, all materials used, before contact with the matrices containing the hLF 1-11 peptide, were subjected to treatment with a 0.05%v/v Tween^®^ 20 water solution to avoid unspecific surface adsorption by the peptide, given that it has been reported that Tween^®^ 20 appears to absorb reversibly on very hydrophobic surfaces and to form a removable layer [49].

**Figure 4:**
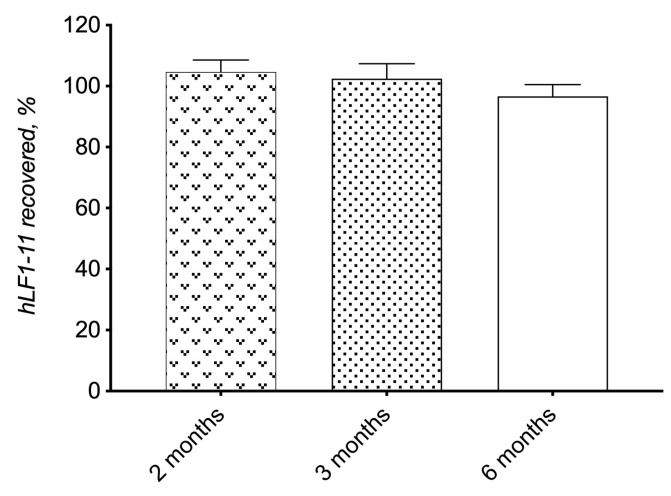
Chemical stability data of HPMC/T2/HA/hLF 1-11_fd_. Data represent mean ± standard error (*n* = 3).

Moreover, a long-term drug stability study was performed on the selected formulation HPMC/T2/HA/hLF 1-11 by monitoring the change in drug recovery up to 6 months. The analysis of drugs in the samples was performed by RP-HPLC, which allowed for the detection of eventual impurities due to the degradation phenomenon. As shown in Figure 4, the hLF 1-11 peptide amount of the HPMC/T2/HA/hLF 1-11 matrix remained above 96% (96.68 ± 3.86%) after 6 months of storage, which is a good result since protein-like drugs are labile to changes of pH and temperature and to oxidation phenomena that can also produce conformational changes in the protein molecule with a loss of antimicrobial activity [29]. Suitable formulations and, in the case of solid formulations obtained by lyophilization, suitable excipients, such as trehalose, need to be used for a long-term storage of protein-like drugs.

#### 3.2.2. In Vitro Susceptibility Testing

MIC and MBC of hLF 1-11 against *S. epidermidis* was 1 μM, showing potency and bactericidal properties of the peptide.

**Figure 5:**
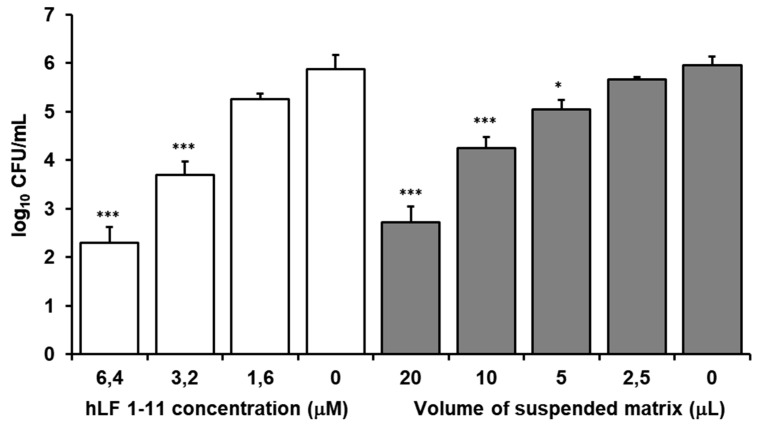
Activity of hlF 1-11 against *S. epidermidis*: cell viability after 1 h of incubation at 37 °C. Activity of the peptide alone at different concentrations and of the peptide released by different volumes of matrices. Statistical significance in each group in comparison with the bars without peptide (Mean ± SE, *n* = 5); (*** *p* ≤ 0.001, * *p* ≤ 0.05).

The inhibitory activity of *hLF1-11* and the release of hLF 1-11 from the HPMC/T2/HA/hLF1-11_fd_ matrix were assessed by an *S. epidermidis* ATCC 35,184 killing assay. As shown in Figure 5, hLF 1-11 released from the matrix exerted high activity against *S. epidermidis*, which was comparable to hLF 1-11 solutions. Notably, a volume of 20 µL of the suspended matrix exerted the same antibacterial activity of a 6.4 µM solution of hLF 1-11, which resulted in a statistically significant 4-log reduction of cell viability. These results indicate that, when released from the matrix, hLF1-11 induces a dose-dependent reduction of *S. epidermidis* viability, demonstrating that the matrix maintains the antimicrobial properties of the peptide unaltered. This result appears relevant, since degradation of peptides can occur during release from delivery systems. When the solid hLF 1-11 is released from the matrix in biological fluid, it absorbs water. The polymeric matrix produces a viscous gel through which the peptide has to diffuse, but aggregation phenomena inside the device can also happen, affecting peptide release and bioavailability. Similar results were obtained using formulations prepared 1, 2, 6, and 15 months in advance.

Therefore, these results highlight the matrix’ capacity to release the peptide while preserving its antimicrobial activity, which is also maintained during a long-term storage of 15 months, underlying the ability of the matrix to maintain the original structure of the hLF 1-11 peptide.

#### 3.2.3. In Vitro Drug Release Experiment

**Figure 6:**
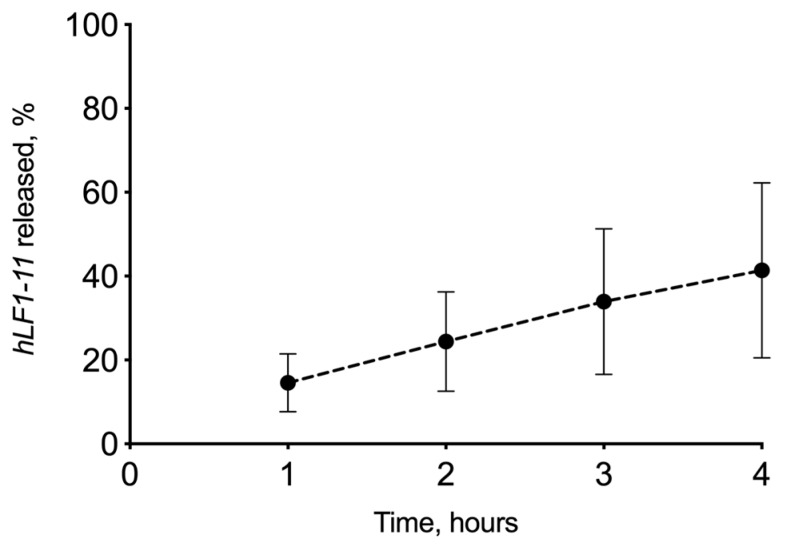
In vitro release profile of *hLF 1-11* in phosphate buffer saline (PBS) from the freeze-dried matrix HPMC/T2/HA/hLF 1-11 containing a hydroxypropilcellulose (22.43 wt %) hyaluronic acid (2.49 wt %), trehalose (49.8 wt %), mannitol (24.92 wt %), and hLF 1-11 (0.33 wt %). Data represent mean ± standard error (*n* = 3).

HPMC/T2/HA/hLF 1-11_fd_ was also tested for preliminary in vitro release studies. In Figure 6, the drug-release profile from the lyophilized matrix is shown. The amount of hLF 1-11 peptide released was 11.71 μg ± 5.44 (SE), about 40%, in 4 h of experiment. From these preliminary data, it is evident that a high variability of the obtained results as demonstrated by the high SE that could be attributed to unspecific adsorption of the peptide on the glass surfaces, even if previously treated with the surfactant solution. Nevertheless, the matrix has shown the ability to release the peptide in a simulated physiological environment as was previously demonstrated by the susceptibility test results.

## 4. Conclusions

The research in the field of antimicrobial peptides and novel approaches for the treatment of infections has mainly focused on identifying more powerful and selective AMPs. However, a deeper knowledge on the delivery of these molecules, considering the problems associated with an efficient and safe release of AMPs, is essential. In fact, with an appropriate drug-delivery system, it is possible to obtain a reduction of the chemical or biological degradation of the AMPs, either in the formulation or after administration, and decrease adverse side effects and control the AMPs’ release rate.

Different vehicles have been developed for the administration of peptides; newer ones are systems that exploit metallic or polymer nanoparticles or microgels/nanogels or surfactant self-assembling systems to control peptide exposure and release as well as to protect incorporated peptides from degradation. Although there are good opportunities for success, numerous issues still remain poorly understood, especially regarding the factors that control the load of the peptide into and the release from the various drug-delivery systems [50].

Since an optimal vehicle does not seem to have been found yet, in this study, new solid matrices containing mucoadhesive polymers obtained by freeze-drying were prepared and characterized. The formulation HPMC/T2/HA/hLF 1-11_fd_ was selected for the delivery of the hLF 1-11 since it showed good drug recovery and no chemical degradation for up to 6 months (long-term stability). Furthermore, the HPMC/T2/HA/hLF 1-11_fd_ matrix allowed for the release of the drug in a simulated physiological environment, linked to an optimal hydration time, and the peptide antimicrobial activity was preserved for up to 15 months of storage. This is a very promising result considering the chemical liability of proteinaceous material. For its properties, the freeze-dried matrix developed in this study could be a good platform for the delivery of antimicrobial peptides in the precorneal area to treat infectious phenomena of the ocular surface. These polymers were chosen on the basis of the results obtained by Burgalassi et al. 2018 [31] who had tested different polymeric materials for the development of solid dosage forms obtained by freeze drying to deliver bevacizumab in the posterior segment of the eye. They evaluated the effect of different semi-synthetic and synthetic products (hydroxypropylmethylcellulose, polyvinyl alcohol, polyvinylpyrrolidone, polyacrylic acid) on hydration time, drug release, and drug stability. Their target, the ocular posterior segment, differed from that of this work (precorneal area). Based on this first evaluation, a selective choice was made to use a biodegradable polymer, hydroxypropylmethylcellulose (HPMC), alone or in combination with hyaluronic acid (HA), known for its mucoadhesive properties, to produce a matrix with suitable hydration and mucoadhesive effects that could improve the peptide interaction with the ocular surface, preventing rapid elimination of the formulation from the site of action and the natural elimination of the polymers.

Moreover, the freeze-drying technology allowed us to obtain a peptide-loaded solid device, overcoming, for example, the alteration of the proteinaceous material following the use of other matrix manufacturing processes, such as the solvent casting, where high temperature or mechanical impacts could destroy the peptide structure.

Emerging technologies represent an opportunity to regulate peptide release and minimize the need for repeated administrations. This research opens new perspectives for ocular topical administration of antimicrobial peptides, providing the basis for technological and biopharmaceutical optimization of the developed formulation.

The formulation could be improved to provide a combined therapy, releasing more than one drug at the same time. In addition, its texture and design could be modified to facilitate administration with the aid of a device, since freeze-dried materials are fragile and may be damaged during manipulated by the patient. Combination therapy might represent a promising option, as synergistic effects would allow for the use of lower doses of both compounds, thus reducing the frequency of emergent drug-resistant strains. Further studies will be performed to evaluate whether the previously described synergistic effects obtained by combining hLF 1-11 with various antibiotics [16] or antifungals [13] could show similar effects after matrix loading, thus allowing prolonged release of synergistic drugs.

## Figures and Tables

**Table 1:** Composition (wt %) of the polymeric vancomycin hydrochloride (VA)-loaded matrices used in this study.

Formulation	HPMC	HA	Mannitol	Trehalose	VA
HPMC/VA_fd_	24.92	-	74.75	-	0.33
HPMC/HA/VA_fd_	22.43	2.49	74.75	-	0.33
HPMC/T2/VA_fd_	24.92	-	24.92	49.83	0.33
HPMC/T2/HA/VA_fd_	22.43	2.49	24.92	49.83	0.33

**Table 2:** Analytical methods specifications.

Drug	Detector	Column	Analysis	Mobile Phase	Flow Rate, mL/min	λ, nm	LOQ ^1^, μg/mL	RT, min
VA	UV	Kinetex Phenomenex^®^ C18 100 Å (5 μm; 150 × 4.6 mm)	Isocratic	NaH_2_PO_4_/Na_2_HPO_4_ (40:60, pH = 6.95, 50 mM)	1.0	210	0.025	5.0
hLF 1-11	DAD	Phenomenex^®^ Aeris widepore XB-C8 200Å (3.6 μm; 150 × 4.6 mm)	Linear gradientfrom 5% to 70% solvent A over 25 min	Solvent A (0.05% TFA in acetonitrile); Solvent B (0.05% of TFA in water)	1.0	220	1.0	5.4

^1^ LOQ: limit of quantitation; RT: retention time.

**Table 3:** Freeze-dried matrices weight of two different batches, Mean ± SE, *n* = 6.

Matrix	Theoretical Weight, mg	Batch 1, mg	Batch 2, mg	CV ^1^
HPMC/VA_fd_	6.00	5.77 ± 0.03	5.85 ± 0.02	1.37
HPMC/HA/VA_fd_	6.00	5.80 ± 0.02	5.77 ± 0.04	1.44
HPMC/T2/VA_fd_	6.00	5.65 ± 0.09	5.67 ± 0.02	2.04
HPMC/T2/HA/VA_fd_	6.00	5.63 ± 0.02	5.73 ± 0.04	1.65

^1^ CV, coefficient of variation.

**Table 4:** Apparent viscosity values before and after the freeze-drying process, Mean ± SE; *n* = 3.

	Viscosity_app_ (mPa*s)	
Polymeric Dispersion	Before	After
HPMC/VA_disp_	227.7 ± 11.82	212.9 ± 10.27
HPMC/HA/VA_disp_	528.3 ± 2.00	351.7 ± 23.76 ^*^
HPMC/T2/VA_disp_	265.2 ± 10.66	275.1 ± 4.42
HPMC/T2/HA/VA_disp_	321.2 ± 5.53	274.8 ± 11.13

^*^ Significantly different value from initial dispersion, *p* < 0.05 (one-way ANOVA; Bonferroni test for multiple comparison).

**Table 5:** VA recovery of the formulations under study (Mean ± SE, *n* = 3).

Matrix	Recovery (%) ± SE
HPMC/VA	92.46 ± 5.09
HPMC/HA/VA	91.39 ± 5.88
HPMC/T2/VA	96.02 ± 2.21
HPMC/T2/HA/VA	100.2 ± 0.16

**Table 6:** hLF 1-11 recovery values from the selected formulations (Mean ± SE, *n* = 3).

Matrix	Measured Concentration (μg/mL)		
	Before Freeze-Drying	After Freeze-Drying	Recovery (%)
HPMC/HA/hLF_fd_	6.96 ± 0.38	4.77 ± 0.31	68.38 ± 4.43
HPMC/T2/HA/hLF_fd_	13.24 ± 1.85	11.99 ± 0.93	86.74 ± 1.83
